# Students’ drinking behavior and perceptions towards introducing alcohol policies on university campus in Denmark: a focus group study

**DOI:** 10.1186/s13011-016-0060-7

**Published:** 2016-04-29

**Authors:** Eva Ladekjær Larsen, Gitte Andsager Smorawski, Katrine Lund Kragbak, Christiane Stock

**Affiliations:** Unit for Health Promotion Research, University of Southern Denmark, Odense, Denmark; University College South Denmark, Kolding, Denmark

**Keywords:** Alcohol, University students, Policy development, Qualitative research, Drinking behavior

## Abstract

**Background:**

High alcohol consumption among university students is a well-researched health concern in many countries. At universities in Denmark, policies of alcohol consumption are a new phenomenon if existing at all. However, little is known of how students perceive campus alcohol policies. The aim of this study is to explore students’ perceptions of alcohol policies on campus in relation to attitudes and practices of alcohol consumption.

**Methods:**

We conducted six focus group interviews with students from the University of Southern Denmark at two different campuses. The interviews discussed topics such as experiences and attitudes towards alcohol consumption among students, regulations, and norms of alcohol use on campus. The analysis followed a pre-determined codebook.

**Results:**

Alcohol consumption is an integrated practice on campus. Most of the participants found it unnecessary to make major restrictions. Instead, regulations were socially controlled by students themselves and related to what was considered to be appropriate behavior. However students were open minded towards smaller limitations of alcohol availability. These included banning the sale of alcohol in vending machines and limiting consumption during the introduction week primarily due to avoiding social exclusion of students who do not drink. Some international students perceived the level of consumption as too high and distinguished between situations where they perceived drinking as unusual.

**Conclusion:**

The study showed that alcohol is a central part of students’ lives. When developing and implementing alcohol policies on campus, seeking student input in the process and addressing alcohol policies in the larger community will likely improve the success of the policies.

**Electronic supplementary material:**

The online version of this article (doi:10.1186/s13011-016-0060-7) contains supplementary material, which is available to authorized users.

## Background

High alcohol consumption among university students is a well-researched health concern in many countries [1]. While most studies have addressed the issue in American student populations [2], several studies are now also conducted in different European countries [3–7]. Results from the Cross National Student Health Survey (CNSHS) conducted in six European countries and Turkey have shown that students in Denmark have the lowest prevalence of abstaining from alcohol when compared to the other countries and that 23 % of male and 8 % of female students showed problem drinking [8]. Heavy alcohol consumption can cause injuries, traffic accidents, assaults, academic problems, and alcohol problems later in life [9–12]. Research evidence suggest that alcohol policies, such as increasing the prices of alcohol and restricting its availability, may be effective in reducing alcohol consumption among students [13–15]. However the implementation of policies is lacking behind; e.g. only a limited number of US campuses have implemented such policies [16] and a survey among leaders of 569 colleges showed that only 30 % have prohibited alcohol consumption on campus [17].

In Denmark, there is a legal framework that prohibits selling any alcohol to persons under the age of 16 and selling strong liquor to persons under the age of 18, but apart from these age limits there are no legal restrictions for selling and using alcohol at universities. In line with the lack of restrictions by law, most Danish universities do not have their own explicit rules that restrict the use of alcohol. In fact, many university events actively encourage the use of alcohol. More specifically, the introduction arrangements for freshmen are frequently centered on alcohol consumption and are officially labeled as “getting drunk tours”. However, there is a growing concern that the strong link between social arrangements and alcohol might lead to exclusion of students who abstain from alcohol for religious, cultural, or health reasons.

Evidence from policy studies show that it is crucial to have knowledge of the societal context and the social setting in which a policy is implemented [18]. We also know that peers play an important role in shaping drinking behavior particularly during years of youth [19]. In Denmark the average alcohol intake for persons above 14 years of age is 11, 1 liters of pure alcohol per year, which corresponds to approximately 14 units of alcohol per week (one alcohol unit = 15 ml pure alcohol) [20]. 20,6 % of the Danish population consume alcohol above the low risk consumption level (14 units of alcohol for male and 7 for female) and 8,3 % consume alcohol above the high risk level (21 units of alcohol for male and 14 for female). A group that stands out is youth enrolled in education where 19 % are at a level of high risk [21]. Although these numbers have decreased since 2010, Denmark has a low proportion of people who abstain from alcohol, a high level of binge drinking, and only small differences in socio-economic status regarding alcohol consumption [22]. Overall, Denmark has a strong tradition of alcohol consumption that is rooted in social and cultural practices [23, 24]. In this liberal culture of alcohol consumption, it may be challenging to successfully implement alcohol policies at universities.

There is limited research that addresses if and how students support campus policies regulating alcohol consumption in Denmark. A survey conducted in 2005 among 548 students from the University of Southern Denmark has shown that less than one quarter of students support a ban of selling alcohol, while the support for such restriction of availability was much higher at six other European universities [8]. This survey study also revealed that female students and those who consumed alcohol at a lower level were more likely to support a restrictive alcohol policy. Although a number of qualitative studies address the drinking behavior of adolescence, no qualitative studies to our knowledge relate drinking behavior to students’ support of alcohol policies on campus. Our aims are therefore to explore this relationship and in particular how students’ drinking behavior shape perceptions towards on campus alcohol policies.

## Methods

### Description of the settings

The University of Southern Denmark (SDU) has more than 27,000 students where approximately 16 % are international students. SDU campuses are located in six different cities in the Southern part of Denmark. Students recruited for this study are enrolled at Odense campus which is the largest campus with 20,675 students (2,332 international students) and at Esbjerg campus, the smallest campus with 1,321 students (431 international students). There are no live-in facilities on the campuses, but apart from being an academic learning environment, the campuses are settings where many social events take place. On Fridays the campus bar is open and is run and organized by students themselves. Other occasions where alcohol is consumed on campus are e.g. graduations, celebrations of exams and events such as sports competitions, music concerts and parties that mark the end or the beginning of a semester. Beer is also available on a daily basis and sold in vending machines and in the canteens. The campuses are also work places for academic and administrative staff, kitchen and cleaning personal, maintenance etc., and serve as venues for receptions, PhD defenses and similar events where alcohol often is served.

A high level of alcohol consumption characterizes the introduction week where students start at the university and are introduced to their programs and fellow students. There is a growing concern that the intense alcohol use neglects the academic content of the week. In 2014 an internal regulation was formed stating that:“*alcohol consumption should never be in the centre of the introduction arrangements*, *but considered as an individual and self*-*determined choice*” [25]. When the study took place (2013–4) this regulation had not yet been implemented and only 2 out of 5 faculties had a policy of limiting the alcohol intake during the introduction week. There are no other alcohol policies at SDU.

### Study design

The study is part of the international informal research initiative “on Campus Alcohol Policy (oCAP)” which aims to explore students’ perspectives of on campus alcohol policies in Slovakia, Belgium, Hungary, France and Denmark. Results from Slovakia have been published earlier and it is intended to compare the results across the participating countries [26]. The data collections and analyses in all participating countries follow the same design, which has been developed jointly in the research group. This comparative research is still ongoing and in this article we report exclusively the results from data collected in Denmark.

To accommodate the aim of the study we conducted focus group discussions (FGD), which is an appropriate method to gain access to the concepts and norms that form participants’ experiences [27]. The advantage of FGDs compared to individual interviews is that it generates data based on group dynamics and is therefore richer in detail than individual interviews [28]. For example discussions of a specific concept are likely to vary since more people express their point of view and at times even disagree on a certain topic.

The richness of the data generated from FGDs depends on how well the group dynamic is working and is related to how comfortable participants feel with one another to engage in the discussion. It has therefore been argued that the composition of the focus groups should be homogeneous [29]. On the other hand, a group that is too homogenous is less likely to provide the richness described above since participants tend to agree with each other. Furthermore, if participants know each other too well, existing hierarchies and leadership may influence what is being said and by whom [28]. Therefore we aimed to invite participants who did not know each other beforehand and varied according to age, gender and study program. Six focus groups were conducted with a total number of 41 participants (Table 1).

**Table 1:** Focus groups

FGD	Danish/International students	Campus	N (total 41)	Gender m/f (total 12 m; 29 f)
1	Danish	Esbjerg	8	4 f, 4 m
2	Danish	Odense	7	7 f
3	Danish	Odense	6	5 f, 1 m
4	Danish	Esbjerg	6	6 f,
5	International	Esbjerg	8	3 f, 5 m
6	International	Odense	6	4 f, 2 m

Recruitment of participants was done by invitation on the university’s intranet and by approaching students in their campus environment. In order to ensure variety between the groups, we recruited students from two different campuses: Odense and Esbjerg. To explore whether there were differences related to national backgrounds, two focus groups were comprised of non-Danish international students. Although the number of participants in this study is small and therefore no definite differences can be stated, it allowed us to contrast statements by Danish and non-Danish students respectively. The majority of participants are female which may be considered a limitation to the study. The unequal gender composition may have had an influence on the group dynamics as well as there may be gender differences related to the topic of investigation [8]. All participated on a voluntary basis and none were excluded from participation (Table 2).

**Table 2:** Characteristics of the participants

FGD/Participant	Gender M/F	Age	Study program	Bachelor/master student	Danish/International student (for the sake of ensuring participants’ anonymity nationality is not specified)
FGD1/1	F	22	Sociology and culture studies	Bachelor student	Danish
FGD1/2	M	22	Sociology and culture studies	Bachelor student	Danish
FGD1/3	F	22	Sociology and culture studies	Bachelor student	Danish
FGD1/4	M	24	Economics and business administration	Bachelor student	Danish
FGD1/5	F	21	Sociology and culture studies	Bachelor student	Danish
FGD1/6	F	22	Sociology and culture studies	Bachelor student	Danish
FGD1/7	M	21	Sociology and culture studies	Bachelor student	Danish
FGD1/8	M	24	Sociology and culture studies	Bachelor student	Danish
FGD2/1	F	20	Law	Bachelor student	Danish
FGD2/2	F	24	Spanish	Bachelor student	Danish
FGD2/3	F	24	International marketing Communications	Bachelor student	Danish
FGD2/4	F	20	Philosophy	Bachelor student	Danish
FGD2/5	F	21	Intercultural pedagogics/Arabic	Bachelor student	Danish
FGD2/6	F	20	Spanish	Bachelor student	Danish
FGD2/7	F	20	Political science	Bachelor student	Danish
FGD3/1	F	23	International marketing communications	Master student	Danish
FGD3/2	F	23	Sports science	Bachelor student	Danish
FGD3/3	F	23	Psychology	Master student	Danish
FGD3/4	F	N/A	Spanish	Bachelor student	Danish
FGD3/5	F	25	Sports science	Bachelor student	Danish
FGD3/6	M	22	Philosophy	Bachelor student	Danish
FGD4/1	F	24	Public health	Master student	Danish
FGD4/2	F	23	Public Health	Bachelor student	Danish
FGD4/3	F	22	Public health	Master student	Danish
FGD4/4	F	22	Environment and ressource management	Bachelor student	Danish
FGD4/5	F	22	Environment and ressource management	Bachelor student	Danish
FGD4/6	F	27	Marketing and innovation	Master student	Danish
FGD5/1	M	25	Sport and event management	Master student	Belgium
FGD5/2	M	26	Public health	Master student	Uganda
FGD5/3	F	25	Business administration	Master student	Bulgaria
FGD5/4	M	24	Public health	Master student	India
FGD5/5	F	27	Public health	Master student	Norway
FGD5/6	M	24	Business administration	Master student	Bulgaria
FGD5/7	M	29	Environment and ressource management	Master student	Cameroon
FGD5/8	F	25	Public health	Master student	Lithuania
FGD6/1	F	25	Brand management &marketing	Master student	Germany
FGD6/2	F	24	Human ressource management	Master student	Italy
FGD6/3	F	26	Cand negot	Master student	Austria
FGD6/4	M	26	Public Health	Master student	Nepal
FGD6/5	M	N/A	International security	Master student	Estonia
FGD6/6	F	26	Master in comparative public policy and welfare	Master student	Romania

Two research assistants, who were graduate students themselves, facilitated the FGDs. They ensured that all participants were included in the discussion by encouraging those participants who were less talkative. The research assistants’ previous roles as students was beneficial as the concepts and language chosen were familiar to the participants [30].

The FGDs followed an interview guide developed by the oCAP research network, which was translated into Danish by two researchers. The guideline contained open statements such as: “Some students frequently drink a lot of alcohol. What is your reaction to this?” The statements served as opening the discussions and it was encouraged that participants during the discussions were given the opportunity to add comments and insights on the topic, which were not included in the interview guide, but nevertheless important to the participants (Table 3).

**Table 3:** Interview guide

Main theme of the discussion	Sub questions
“Some students frequently drink a lot of alcohol”. What is your reaction to this statement?	What are your own experiences? Explain why this statement is true or false according to you. In what way can alcohol use be a problem to students? Which problems can arise from student alcohol use? What is your view on problematic alcohol use? Are there a lot or few students that use alcohol problematically? Can you explain why?
Which possible problems could occur on your campus because of the use of alcohol and/or the effects of alcohol use?	Which problems have you encountered yourself? What are according to you the most important problems? Which problems are of lesser importance? Who might be affected/influenced by these problems? Which influence do these problems have on you? (*in case of* “*there are no problems*”) Which problems concerning alcohol use could hypothetically happen on your campus? If these problems were to happen, to what extent are they important to you?
Which rules/agreements or policy are there on your campus concerning the use of alcohol and the effects of alcohol use?	Which rules/agreements are there on the use of alcohol on your campus? How are these rules/agreements made public? Who do these rules apply to? Who has constructed these rules? For which reason are these rules being followed? For which reasons are they not being followed? What are the positive aspects of these rules concerning alcohol use on your campus? What are the negative aspects of these rules concerning alcohol use on your campus? Which adjustments should be made to these rules/agreements and/or this policy, according to you? (*in case of* ‘*no rules*’) What is your opinion about this lack of rules? What is positive about this situation? What is negative about this situation?
Let us brainstorm about this. Imagine that you were the head of campus and you could develop an on campus alcohol policy. Which rules/agreements concerning alcohol use and/or the effects of alcohol use should there be on your campus?	Which rules/agreements concerning the use and/or effects of alcohol would you like at your campus? Which rules do you think are more important? Which are less important? For what reasons? Which side-conditions should be fulfilled when implementing these rules? What purpose should these rules have? Who should be involved in the development of these rules? Who should be involved in the implementation of these rules? To whom should these rules apply? Which problems might arise in making/implementing/… these rules? In which way should compliance to these rules be controlled? In which way should violations be sanctioned? What would you do if someone does not follow these rules? (*In case of* ‘*there are no problems*’) In which way can possible problems concerning alcohol use be prevented? Is setting rules one of them? Do you think it is meaningful/useful to set rules on campus alcohol use? Explain why/why not.
If I summarize, then … (give summary of main points that were discussed, appointments that have to be made) with a few side-conditions… (give summary of side-conditions)	Imagine that these rules were to be implemented starting tomorrow morning: what will be the effect? In what way can the implementation of these new rules solve existing problems concerning alcohol use at your campus? Which, if any, problems will not be solved by implementing this new policy? Can these problems at all be solved through rules and regulation?
We have discussed a lot of things today, which we thank you for. We are confident that your opinion has given us a better insight on the issue. Is there anything more you would like to share with us, that was not talked about during this discussion	

### Analytical procedure

All focus group discussions were digitally recorded and transcribed verbatim by the research assistants. The analysis was based on a thematic approach and followed a codebook developed by researchers from the oCap network (see also Additional file 1). The codebook was developed a priori from existing concepts that we wished to apply to the analysis and consisted of predetermined categories, codes and sub-codes. Relevant passages from the transcripts were added to the codebook under the corresponding codes and sub-codes. This made sure that the data could be compared across the various transcripts [31]. While it was crucial to ensure analytical consistency, since many researchers and from different nations were involved in the project, it was likewise crucial to ensure that new categories could evolve based on the data [32]. The analytic phase was therefore an iterative process that allowed reduction and simplification of the dataset, while making new connections between the concepts and reconceptualizing preexisting categories [33]. The data implemented in the code book has therefore been further condensed into three themes: 1) Formal and informal rules and responsibility, 2) Alcohol as part of student life, and 3) Alcohol use during the introduction week (see also Tables 4, 5 and 6). The identification of these themes can be characterized as an axial coding process where meaningful units were identified and connection between the codes were compared across the entire code book [34].

**Table 4:** Development of themes

Theme 1	Formal and informal rules and responsibilities			
Category	Rules and policy	Responsibility for alcohol use		
General code	- Rules on the campus - Attitude towards rules on campus - Proposals for rules on the campus - Attitude towards rules on the campus - Proposals for prevention by the university for alcohol related problems - Attitude towards proposals for prevention by the university for alcohol related problems - Stakeholders - Policy is necessary and meaningful - Goal of policy - Effect of Policy	- University is responsible - Students are responsible themselves - Social control is responsible - Fraternities–student clubs are responsible

**Table 5:** Development of theme 2

Theme 2	Alcohol as part of student life	
Category	Situation alcohol use	Alcohol related problems
General code	- Alcohol use among students in general - General attitude towards alcohol use - Alcohol use is part of a student’s life - Prevalence of alcohol use - Alcohol events–situations - Drunkenness	- Alcohol related problems for yourself - Alcohol related problems for others - On campus alcohol problems - Problematic alcohol use - Prevalence alcohol problems

**Table 6:** Development of theme 3

Theme 3	Alcohol use during the introduction week			
Category	Rules and policy	Responsibility for alcohol use	Situation alcohol use	Alcohol related problems
General code	- Rules outside of campus - Attitudes towards rules outside of campus - Proposals for rules outside of campus - Attitude towards proposals of rules outside of the campus - Proposals for rules on the campus - Proposals for prevention by the university for alcohol related problems - Attitude towards proposals for prevention by the university for alcohol related problems - Stakeholders - Policy is necessary and meaningful - Goal of policy	- Social control is responsible	- General attitude towards alcohol use - Alcohol use is part of a student’s life - Prevalence of alcohol use - Alcohol events–situations - Drunkenness - General attitudes towards on campus drinking	- Alcohol related problems for others - On campus alcohol problems

### Ethics

All participants signed informed consent and were guaranteed anonymity in all published and presented material.

## Results

### Alcohol as a part of student life

Students expressed that starting at the university is also a time where they are concerned with making new friends. Usually it would involve drinking alcohol, particularly during the beginning of one’s study: “*The first semesters were definitely characterized by going out*, *both on Thursdays*, *Fridays and Saturdays*” (FGD2/2.) There appeared to be consensus among the students that partying decreased as the studies progressed. Still, alcohol was an integrated practice when celebrating an exam and when social arrangements were held on and off campus:

FGD1/8: In the beginning of a semester you see many students who encourage you to drink like: “come on let’s go to the bar”. The closer you get to an exam period the less you go out.

FGD1/4: Yeah and right after an exam you go to celebrate.

FGD1/7: It is a kind of feeling of togetherness. When we party we all do it together. It is not like you two and two take a beer. It is like the whole class together goes crazy for a month or so. But the most important problem is that some are socially excluded.

Interviewer: When they drink or?

FGD1/4: When they don’t drink

FGD1/6: Especially after a weekend if for example three people in a group and two of them sit and talk about their parties. Then an hour will pass sharing stories but the person who didn’t party has nothing to share.

The *feeling of togetherness* was an important aspect of drinking, particularly during the beginning of one’s study where students did not know one another. The discussion of the amount and frequency of drinking was also discussed in other groups and was here associated to the unstructured daily life that students had:

FGD3/2: I think it is individual how much and when you drink. To me it is nice just to meet with a few friends and share a bottle of wine and that is not considered by others to be a negative thing. But one bottle can of course become two.

FGD3/3: I think as a student it is easy to drink a lot because we have a very unstructured daily life. Sometimes we only have two lectures per week and then it is easy to have a party. But once we join the working life I think it will be different.

FGD3/5: I think it is different according to your study program. On sports science I don’t think we drink so much compared to what I hear of in other study programs. I think it depends on what kind of daily life you have. If you have classes from 8 to 16 and you have to do sports for 5 hours you don’t just grab a beer during breaks.

Overall students shared the opinion that the level of alcohol consumption was high, but not necessarily problematic. Drinking behavior was perceived as a central element of Danish culture and students did not consider their own alcohol consumption as particularly different from the majority of Danes: “*Well*, *now here in Denmark and generally in the Danish culture it is so*, *that if you are going to be social*, *then alcohol works as a kind of social lubricant*” (FGD1/8). Drinking was also considered as something you become socialized to do during early teenage years: “*It is also a norm you bring from high school. If you did not drink you were looked upon as a social outcast*” (FGD1/4). Other students referred to that their parents often shared a bottle of wine at dinner and that they were brought up with alcohol present at family parties and other social gatherings.

The groups comprised of international students expressed that they were surprised to meet a university setting where alcohol was an integrated practice on campus and where there were no regulations. But like the Danish students, the international students also stressed that alcohol consumption was a central part of Danish culture: “*I see the consumption of alcohol on campus as a part of a bigger picture. Generally in Denmark there should be less alcohol consumed. And then of course*, *the rest will come from that. I would not consider a written policy here effective*” (FGD 6/1).

But some international students perceived the level of consumption as too high and distinguished between situations where it was unusual to drink in their perspective: “*Okay*, *you have a sport event*, *and the purpose is who is getting drunk as the first. I was really shocked. Yeah it is something fun*, *something cool*, *but I related sport to something healthy*” (FGD 5/3).

Although both groups of international students agreed that there was a high level of alcohol consumption compared to what they were used to, they also argued that some international students became socialized to adapt this behavior: “*From Wednesday to Friday in three different bars in the city you can get free beer. The internationals that come here*, *they go crazy*, *because it is for free*” (FGD 6/3).

### Rules, regulations and responsibility of drinking

When discussing existing alcohol policies and rules on campus the students expressed that there are not many rules in the university setting. Generally, they and university staff perceived students as adults who are responsible for their own actions: “*We are adults and that is what we have been told from the beginning. Now we are students and it is our own responsibility to go through this study in best possible ways. And then we should also control other things* [drinking and partying]. *That is the attitude we have been introduced to*” (FGD 2/5). This issue of responsibility was also brought up in FGD 1:

FGD1/6: About rules and policies they are non-existent. And that is actually funny, I heard of two instructors they were sitting at a post [during the introduction week instructors, who are older students, arrange competitions for the new students] drinking beer. And then new students said: “What the fuck are you drinking? beer?” and they answered: “sure and you can just go and buy if you like. You are grown ups. You are at the university”.

FGD1/3: I also asked the first time I came here. There was an arrangement at the bar during the intro days and there was a tutor drinking. And I asked:” Can you drink everywhere here? Can I just open a beer anywhere”. I was kind of surprised that it was legal.

FGD1/3: Yes and you can buy beers in the canteen.

Several students found restrictions of alcohol consumption on campus unnecessary: “*I don*’*t see it as a big problem here*, *so I don*’*t think we need restrictions*” (FGD 5/5). There were also students who found that making rules would only create problems: “*I do see the point of making a rule that you are not allowed to drink while in class. That makes sense. But why make that rule if there is no problem*? *By making that rule*, *you create a problem*” (FGD 1/7).

There were some suggestions concerning limiting the accessibility of alcohol. For example some students thought it was unnecessary to sell alcohol in the canteen and in the vending machines, while others thought it was fine:

FGD4/6: I think it is good because every time we finish something and have passed an exam we have been very nervous about then we celebrate with a glass of champagne. But I think that people are reasonable enough to be able to control it.

FGD4/1: I think the reason why there is no alcohol policy is that perhaps there haven’t really been any problems.

[Other group participants are nodding to express this point of view]

Other groups discussed that some areas on campus or some situations could ban alcohol intake, but again was associated to that it was not really necessary since it was common sense not to drink for example in the library or during lectures:

FGD 5/5: I think it can be like some general rules like it is on the library and not drink in the lectures, that sort of thing. But I don’t see it as a big problem here, so I don’t think we really need restrictions.

Interviewer: Okay.

FGD5/6: I think there shouldn’t be any restrictions. I mean, we’re missing the points. We shouldn’t treat students like mindless teenagers of 15 or 16 years old. Nobody will go to a lecture drunk, that’s just stupid […]. Because if you forbid something or impose some restriction, you … I mean how the human nature move, you make it more desirable. But having no regulations at all mean we could just trust the students not to behave in inappropriate matter. Of course they could that, but not because of our coping, because of something else. That is what the guides or rules should say. You shouldn’t behave in inappropriate matter regardless of what the reason is.

FGD5/1: I agree. Because I think if you’re drunk the last thing that comes to your mind is to go to a lecture. You don’t want to go.

[The other participants laugh]

Less common was the attitude that policies were regarded as a mean to change the overall drinking behavior. But some students expressed that a firm alcohol policy could possibly generate a greater professionalism and that more students would perform better.

Overall students were against radical regulations such as a total ban of alcohol, but they did not consider all alcohol related behaviors as appropriate. When discussing the topic of problematic drinking behavior on campus they would first mention the extreme cases of inappropriateness. For example students found drinking alcohol during class inappropriate: “*I remember having a lecture Thursday late in the afternoon where the beach bar was open and people sat with beers in front of them during the lecture. It is lacking a bit of respect in some ways*” (FGD 1/8). Another respondent drank during a lecture and subsequently reflected critically about own behavior and the consequences of it: “*We bought 3 beers and sat down in class and it was stupid because after the first hour the concentration level was gone. We were not present in the room we were already gone off to the weekend. You have to separate these two things*” (FGD 3/6).

Apart from drinking during the lectures participants also found it inappropriate to drink when the consequences of it was that a student no longer was capable to fulfill his/her duties and missed out on classes: “*When it* [drinking] *effects your study when you have been out drinking on a weekday and you think* “*I can easily go to the lectures tomorrow*” *and you then can*’*t when you wake up the morning after*” (FGD 3/3).

It was also considered inappropriate to drink alone and for no particular social reason. Furthermore, being out of control and jeopardizing one’s own health was also considered to be “*out of hand*”: *I have picked up diabetic fellow students from a bush a winter night. This I might see as a problem. When you can no longer control your own disease due to drinking*” (FGD 2/6). Although drinking was described as a large part of students’ lives it was not tantamount to that all and any kind of alcohol behavior was socially accepted. Being influenced by alcohol during lectures was regarded as disrespectful and as not being able to fully understand the content of the class. Moreover risking ones health or drinking alone was problematic. Besides from identifying inappropriate drinking behavior the above statements underline that drinking is something you do for being social and for fun.

### Alcohol use during the introduction week

One situation that stands out as particularly related to heavy use of alcohol is the introduction week. However students acknowledged that alcohol consumption could have negative consequences in terms of social exclusion of those students who do not drink alcohol. At the time of the interview only two out of five faculties had made regulations of alcohol consumption in the introduction week and during the FGDs this topic was highly debated:

FGD 2/7 “My own introduction tour was also without alcohol. I cannot imagine a tour that includes alcohol. So it works quite fine”.

FGD 2/6: Ehh, talking about the introduction week, I think the night where people were drunk we had a lot of games like performance, singing or speaking in foreign languages […..]. That was not a problem for those who were drunk because they had a lot of fun. There was a small group of people who did not drink. It was really unacceptable to them to make such performances. I think that people didn’t think about it. To them it was a natural thing to do it if you were drunk. But to me it is not a natural thing to sing in front of others when I can’t really sing.

Other students stated that an important purpose of the introduction week was to get to know one another and alcohol was therefore a central part. But some students felt under pressure during the introduction week: “*I felt pressured to drink and I ended up having some shots*, *but I did it because I thought*: “*I am in a new place and I don*’*t make any friends if I resign from fun*” (FGD 3/4).

Among participants there was an understanding towards those students who do not drink alcohol. They considered it unfair that these students felt socially excluded and suggestions concerning regulations were made. There was no consensus to completely forbid alcohol, but students were positive towards limiting the intake of alcohol during the introduction week for example by allowing it only at the last day.

Interviewer: At some study programs there is no alcohol.

FGD4/6: that is fucking borrowing [laughs]

Interviewer: I had a discussion with some students who participated with no alcohol. And to them it was not a problem because they did not know of other ways [to have an introduction week]. The ones who complained were the instructors. There was one night where alcohol was allowed but other than there was a lot of sport activities.

FGD4/6: I think that is a bit weird. And what about that person who really sucks at ball games? It is a pity to him too.

FGD4/1: But if more people don’t drink? Many are not strong enough to say no.

FGD4/6: That is how alcohol is. If someone doesn’t want to drink they can sit in the corner and play cards.

[other participants laugh]

FGD4/6: Well, I don’t know. I just think it is wrong because to me alcohol is part of the culture. So I think it would be very wrong to join an introduction tour without alcohol.

Discussing the use of alcohol during the introduction week and introducing alcohol restrictions during this week essentially captured the dilemma of the feeling of togetherness and having fun versus exclusion of those students who did not drink. While some students were in favor of restrictions in order to acknowledge the avoidance of exclusion, other students were against it based on the argument that it should be an individual choice. There appeared to be a pattern that those who supported regulations had already experienced an alcohol-free introduction week or felt excluded and those who were against it experienced alcohol as a ‘natural part’ of the introduction week.

## Discussion

The results illustrated that alcohol consumption is an integrated part of students’ lives. Particularly in the introduction week and during the first study year, alcohol was frequently and heavily consumed. There seemed to be consensus that the pattern of high and frequent levels of alcohol consumption decreased over time of studies. This pattern is much in line with the general alcohol behavior pattern in Denmark, where alcohol consumption in party contexts decreases with age whereas alcohol consumed alone and with family increases with age [35]. High alcohol consumption was related to starting a university education where one would have to make new friends in a new social setting. In that sense alcohol becomes a means to create social bonds and making friendship [36]. Saying no to alcohol may in this sense be similar to rejecting your fellow students. This concern was prevalent among participants in this study who argued that students abstaining from alcohol might have felt socially excluded particularly during the introduction week. It has been argued elsewhere that binge drinking is a mark of youth having reached autonomy from parents and in this sense drinking are ‘rituals of maturing’ [37]. Although university students in our study are ‘older’ youth, and presumably have not been under parents’ influence for a number of years, the drinking culture at university may be linked to a way of practicing youth culture. In addition, it was expressed that students’ drinking behavior is their own responsibility and something that the university should not interfere in.

There were some situations where it was considered inappropriate to drink alcohol. Drinking in class was not a common practice, but was perceived as beyond limits. Being in a learning environment was associated with concentration and academic performance whereas drinking was related to partying and having fun. The contradictions of the meanings attached to these situations may explain why drinking in class was a relatively rare phenomenon. Other non-acceptable situations were situations where students lost control and put themselves at risk, getting drunk alone, or losing their study capability. The characters of these non-acceptable drinking situations highlight that drinking is a practice associated with being social, having fun and making friends.

Although high levels of alcohol consumption is perceived as a natural part of youth and student life, binge drinking at the university should be seen in relation to the drinking culture in Denmark. Both Danish and international students highlighted that binge drinking was not exclusively a practice occurring at the Danish universities, but rather a practice permeating various kinds of social occasions and for all age groups. A recent study of the Danish drinking culture found that in countries where adults have a high level of alcohol consumption, adolescents have been drunk three times more than adolescents in countries where the alcohol consumption among adults is low [38]. The culture of drinking is reproducing itself with new generations adapting and taking over existing habits. This perspective was reflected in our study where students found it useless to only implement alcohol preventive policies at universities and by the finding that international students were socialized to adapt to the drinking culture among their Danish peers. In the opinion of both Danish and international students, a change in drinking behavior would only be likely to occur if interventions were made across different social settings and at a national level. In addition, the participants regarded it unnecessary to make policies and did not experience alcohol as a problem on campus. Some students perceived restrictions as intimidating their personal freedom. On the other hand, there were some suggestions regarding the contents of policies. These were mostly centered on making restrictions during the introduction week and mainly for the reason to avoid exclusion of students who do not drink. This indicates that students are not completely reluctant to accept some regulations. These perspectives are possibly explainable in the light of a beginning change in the Danish drinking culture. Some studies have shown a decrease in the amount of alcohol consumed among Danish adolescents over the last decade [39]. This suggests that a change of drinking norms is taking place, one that possibly will affect the drinking culture at Danish universities in the near future.

## Conclusion

This study explored students’ perceptions of introducing alcohol policies on campus. It was found that alcohol consumption is an integrated part of students’ life and associated with being social and making new friends. Additionally it was found that the high level of alcohol consumption not only occurs at universities, but also was perceived to be a part of overall Danish culture. It could therefore be argued that alcohol policies on campus would not be sufficient alone in order to change the drinking culture.

Although alcohol consumption was perceived as a common practice, students defined problematic alcohol behavior as drinking in social isolation and as losing control. But not being a norm may explain the reluctance towards accepting alcohol policies on campus. On the other hand, there was arguments to decrease the role of alcohol during the introduction week as well as some restrictions in alcohol availability suggesting that students are willing to accept some regulations on campus, if they are sensitive to students’ perceptions and wishes.

## Additional file

Additional file 1: Appendix 1: Code book. (XLSX 20 kb)
